# The efficacy of retrograde intra-renal surgery (RIRS) for lower pole stones: results from 2946 patients

**DOI:** 10.1007/s00345-023-04363-6

**Published:** 2023-03-17

**Authors:** Carlo Giulioni, Daniele Castellani, Bhaskar Kumar Somani, Ben Hall Chew, Thomas Tailly, William Ong Lay Keat, Jeremy Yuen‑Chun Teoh, Esteban Emiliani, Chu Ann Chai, Andrea Benedetto Galosi, Deepak Ragoori, Yiloren Tanidir, Saeed Bin Hamri, Nariman Gadzhiev, Olivier Traxer, Vineet Gauhar

**Affiliations:** 1 Urology Unit, Azienda Ospedaliero-Universitaria delle Marche, Polytechnic University of Marche, Ancona, Italy; 2 Department of Urology, University Hospitals Southampton, NHS Trust, Southampton, UK; 3 Department of Urology, University of British Columbia, Vancouver, Canada; 4 Department of Urology, University Hospital of Ghent, Ghent, Belgium; 5 Department of Urology, Penang General Hospital, Penang, Malaysia; 6 Department of Surgery, Faculty of Medicine, S.H. Ho Urology Centre, The Chinese University of Hong Kong, Hong Kong, China; 7 Urology Department, Fundación Puigvert, Universidad Autónoma de Barcelona, Barcelona, Spain; 8 Department of Surgery, Urology Unit, University Malaya, Kuala Lumpur, Malaysia; 9 Department of Urology, Asian Institute of Nephrology and Urology, Irram Manzil Colony, Hyderabad, Telangana India; 10 Department of Urology, Marmara University School of Medicine, Istanbul, Turkey; 11 Division of Urology, Department of Surgery, Ministry of the National Guard Health Affairs, King Saud Bin Abdulaziz University for Health Sciences, King Abdullah International Medical Research Center, Riyadh, Saudi Arabia; 12 Endourology Department, Saint-Petersburg State Medical University, Saint-Petersburg, Russia; 13 Department of Urology AP-HP, Sorbonne University, Tenon Hospital, Paris, France; 14 Department of Urology, Ng Teng Fong General Hospital, Singapore, Singapore; 15 Department of Urology, Polytechnic University of Marche, 71 Conca Street, 60126 Ancona, Italy

**Keywords:** Nephrolithiasis, Retrograde intra-renal surgery, Lower polar stones, Endourology, Laser lithotripsy

## Abstract

**Purpose:**

To evaluate the perioperative outcomes of retrograde intra-renal surgery (RIRS) for lower pole stones (LPS) and factors affecting stone-free rate (SFR).

**Methods:**

Data from 20 centers were retrospectively reviewed**.** Inclusion criteria were adult patients, normal renal anatomy, and LPS. Exclusion criteria were bilateral surgery, concomitant surgery for ureteral stones. SFR was defined as a single residual fragment (RF) ≤ 2 mm and evaluated 3-months after surgery. A multivariable logistic regression analysis was performed to assess factors associated with RF. Statistical significance was set at *p* value < 0.05.

**Results:**

2946 patients were included. Mean age and stone size were 49.9 years 10.19 mm, with multiple LPS in 61.1% of cases. Total operation and laser time were 63.89 ± 37.65 and 17.34 ± 18.39 min, respectively. Mean hospital stay was 3.55 days. Hematuria requiring blood transfusion and fever/urinary infections requiring prolonged antibiotics occurred in 6.1% and 169 5.7% of cases, while sepsis with intensive-care admission in 1.1% of patients. On multivariate analysis, Multiple stones (OR 1.380), stone size (OR 1.865), and reusable ureteroscopes (OR 1.414) were significantly associated with RF, while Thulium fiber laser (TFL) (OR 0.341) and pre-stenting (OR 0.750) were less likely associated with RF.

**Conclusions:**

RIRS showed safety and efficacy for LPS with a mean diameter of 10 mm. This procedure can achieve a satisfactory SFR in pre-stented patients with a single and smaller stone, particularly with TFL use.

**Supplementary Information:**

The online version contains supplementary material available at 10.1007/s00345-023-04363-6.

## Introduction

Lower pole stones (LPS) account for up to 35% of renal stones, thus being the site with high incidence [1]. The anatomical challenges of infundibular-pelvic angle and long narrower infundibulum contribute to its increased incidence, with a decreased probability of spontaneous expulsion [2] and posing nuances in such stone management. For this reason, the American Urological Association (AUA) guidelines proposed an algorithm of intervention, preferring percutaneous nephrolithotomy (PCNL) for its higher stone-free rate (SFR) in the treatment of LPS larger than 10 mm [3]. However, retrograde intra-renal surgery (RIRS) is considered a valid option thanks to advancements in scope technology, refinement in technical expertise, and the advent of powerful lasers [3].

Since the introduction of the first flexible ureteroscope, technological evolution has led to improvements in several aspects, such as fiber optics digitalization, miniaturization of scope dimensions, development of disposable scopes, and, mostly, scope deflection [4]. Regarding single-use ureteroscopes, their introduction has made RIRS even more attractive for the LPS thanks to scope diameter decrease with, consequently, higher deflection and better use for the lower pole. This had led in reduction of surgical times, and allowed to perform an endoscopic procedure without any fear of damage of expensive reusable scopes [5]. Yet, the use of Thulium fiber laser (TFL) may also play an important role in RIRS, since it was found in preclinical studies to allow for ablation at twice the speed of holmium laser with four times the amount of dust generated [6].

Nevertheless, RIRS for LPS is still challenging due to anatomical factors affecting the scope deflection with laser fiber in the working channel [7]. Moreover, infundibular width and length and infundibular-pelvic angle hinder the feasibility of the procedure and spontaneous passage of fragments, influencing the SFR [8].

In the present study, we aimed to evaluate the efficacy and safety of RIRS for LPS management in a large, multi-center series. The secondary aim was to assess the practice preferences of experienced surgeons in terms of types of scopes, lasers, and lithotripsy techniques when tackling LPS by RIRS.

## Materials and methods

### Enrolment protocol

Worldwide specialists in RIRS were invited to collaborate in the creation of a retrospective registry for adult patients undergoing flexible ureteroscopy (F-URS) for kidney stones. RIRS was performed as per the standard of care and surgical practice of each contributing institute. The FLEXible ureteroscopy Outcomes Registry (FLEXOR) was created as part of an endeavor of the Team of Worldwide Endourological Researchers (TOWER), research wing of the Endourological Society [9]. Twenty centers from fifteen countries joined the FLEXOR project, including 6669 patients undergoing RIRS for renal stones between January 2018 and August 2021. Institutional board review approval was obtained by the Asian Institute of Nephrology and Urology Hyderabad, India (#AINU 08/2022). Each center acquired its ethics board approval before contributing and provided anonymized data. All patients signed informed consent.

### Inclusion and exclusion criteria, and data collection

Inclusion criteria were patients aged ≥ 18 years, with normal renal anatomy and pelvic-calyceal system who underwent only RIRS for renal stones of any size and number and localized in the lower pole. Exclusion criteria were children/adolescents, renal collecting system anomalies, ureteral stones, concomitant bilateral procedures, and stones located in the upper and medium pole, and in the pelvis.

Patients with missing data were also excluded. The following data were gathered: patient clinical characteristics, stone specification (i.e., Hounsfield Units (HU), greatest transverse diameter, and single vs multiple), type of instruments and lasers, lithotripsy technique, intraoperative data, postoperative complications, and SFR. Surgical time was estimated as the time from the start of the procedure to the placement of a bladder catheter. RIRS was done in all centers as per current practice [10].

### Patient follow-up and secondary treatment

Patients were assessed 3 months after surgery according to the standard of care of each participant center, which included KUB X-ray and/or ultrasound or non-contrast CT scan. Stone-free status was defined as the absence of fragments > 2 mm.

Secondary treatment was required in the presence of significant residual fragments (RF), an upper urinary tract obstruction by RF, or if treating physicians judged it necessary. Secondary treatment was performed for fragments deemed significant by treating urologists based on their clinical assessment.

### Statistical analysis

The Kolmogorov–Smirnov test was used to check data distribution for normality. Continuous data are reported as mean and standard deviation. Categorical data are presented as absolute numbers and percentages. A multivariable logistic regression analysis was performed to assess factors associated with RF. Data are presented as odds ratio (OR) and 95% confidence interval (CI). Statistical tests were conducted using the SPSS software package version 25.0 (IBM Corp., Armonk, NY).

## Results

Among patients included in the FLEXOR project, 2946 patients met the inclusion criteria and were included in the analysis. Table 1 shows patient baseline characteristics. There were 1941 (65.9%) males. The mean age was 49.9 ± 15.47 years. The most common symptom of the presentation was pain (57.6%). 399 (13.5%) patients had their stone(s) incidentally found. CT scan was used as diagnostic imaging in 85.8% of cases. Roughly half of the patients were pre-stented before RIRS. The mean stone size was 10.19 ± 8.42 mm and 61.1% of patients had multiple LPS. The mean HU of the largest stone was 971.33 ± 347.61.

**Table 1:** Patients’ baseline characteristics

Characteristic	Numbers
Total number of patients	2946 (100%)
Age, *years*	49.90 (15.47)
< 40	890 (30.2%)
41–65	1583 (53.7%)
66–75	322 (10.9%)
> 75	151 (5.1%)
Gender	
Male	1941 (65.9%)
Female	1005 (34.1%)
Symptoms of presentation	
Hematuria	164 (5.6%)
Pain	1698 (57.6%)
Fever	297 (10.1%)
Elevated creatinine	330 (11.2%)
Incidental findings	399 (13.5%)
Missing	58 (2%)
Pre-operative imaging	
CT scan	2160 (73.3%)
Contrast enhanced CT	369 (12.5%)
X-ray	805 (27.3%)
Ultrasonogram	1436 (48.7%)
Pre-operative stenting	1503 (51.5%)
Stone characteristics	
Stone density, Hounsfield unit	971.33 (347.61)
Multiple stones	1801 (61.1%)
Stone size, mm	10.19 (8.42)

Data are presented as means (standard deviation) and frequencies (proportions)

Table 2 shows intraoperative characteristics. Almost all patients had their surgery done under general anesthesia (93.9%) and in the lithotomy position (99.9%), with the surgical table in a flat position in 71.9% of cases. Total operation and laser time were 63.89 ± 37.65 and 17.34 ± 18.39 min, respectively. Two-thirds of surgeries were performed using holmium laser (78.6%), and a combination of lithotripsy techniques was performed in 64.7% of cases, while basket extraction of fragments was used in 32.8% of procedures.

**Table 2:** Intraoperative characteristic of lower pole stone patient cohort

Perioperative parameters	Numbers
Operation performed by consultant	2201 (74.7%)
Types of anesthesia	
General anesthesia	2766 (93.9%)
Spinal anesthesia	180 (6.1%)
Respiratory control	
None	1490 (50.6%)
Gated respiration	1015 (34.5%)
Apneic	441 (15.0%)
Patients position	
Lithotomy	2942 (99.9%)
Supine with split leg	4 (0.1%)
Table position	
Flat	2118 (71.9%)
Head-up	633 (21.5%)
Head-down	195 (6.6%)
Surgeon position	
Standing	2311 (78.4%)
Sitting	635 (21.6%)
Types of scope	
Reusable	2071 (70.3%)
Disposable	875 (29.7%)
Laser fiber used	
Thulium fiber	630 (21.4%)
Holmium laser	2316 (78.6%)
Stone handling techniques	
Dusting	1703 (57.8%)
Fragmentation	1018 (34.6%)
Combination techniques	1906 (64.7%)
Extraction using baskets	967 (32.8%)
Total laser time, min	27.34 (18.39)
Total operation time, min	63.89 (37.65)
Intraoperative complications	
Pelvicalyceal system bleeding not requiring blood transfusion	184 (6.2%)
Pelvicalyceal system bleeding requiring blood transfusion (Clavien grade 2)	3 (0.1%)
Ureteric injury due to access sheath requiring stenting (Clavien grade 3)	73 (2.5%)

Data are presented as means (standard deviation) and frequencies (proportions)

Supplementary Table 1 depicts postoperative outcomes. The mean hospital stay was 3.55 ± 3.38 days. Hematuria requiring blood transfusion occurred in 181 patients (6.1%). Regarding infectious complications, fever/urinary infections (Clavien grade 2) requiring prolonged antibiotics occurred in 169 (5.7%) patients, whereas sepsis with intensive-care admission (Clavien 4) was diagnosed in 33 (1.1%). There were 73 (2.5%) ureteral injuries requiring prolonged stenting (Clavien grade 3). At 3 months, 654 (22.2%) patients were diagnosed as having RF, and among these 60.8% required further treatment(s).

Multiple stones (OR 1.380 95% CI 1.235–1.542, *p* < 0.001), stone size (OR 1.865 95% CI 1.525–2.282, *p* < 0.001), and the use of a reusable ureteroscope (OR 1.414 95% CI 0.819–2.441, *p* = 0.003) were significantly associated with RF at multivariable analysis (Table 3). Conversely, TFL lithotripsy (OR 0.341 95% 0.252–0.461, *p* < 0.001) and pre-stenting (OR 0.750 95% CI 0.647–0.868, *p* < 0.001) were predictors of SFR.

**Table 3:** Multivariable logistic regression of factors associated with residual fragments after surgery

Parameters	Residual fragments OR (95% CI)	*p* values
Multiple stones	1.380 (1.235–1.542)	< 0.001
Stone size	1.865 (1.525–2.282)	< 0.001
Pre-stenting	0.750 (0.647–0.868)	< 0.001
Thulium fiber laser used	0.341 (0.252–0.461)	< 0.001
UAS used	1.414 (0.819–2.441)	0.214
Reusable scope	1.445 (1.138–1.835)	0.003

## Discussion

RIRS for LPS is one of the most intriguing procedures for renal calculi due to patients’ and stone characteristics that affect its success [11]. New scoring systems have been proposed to select the most appropriate endoscopic treatment to improve the single-stage SFR for 1–2 cm LPS, and the best approach for kidney stones has not yet been determined [12]. Even a three-dimensional software, named Kidney Stone Calculator, was developed to improve surgical planning, estimating the stone volume and lithotripsy duration (DL) [13]. Although the software demonstrated reproducibility and accuracy, LPS showed a higher difference between the expected and actual DL in case of no relocation of the stone.

As most single-center studies have different results on SFR, we endeavor to have a real-life global series on the practice patterns and outcomes in LPS. In the present study, we evaluated the perioperative outcomes of 2946 patients who underwent RIRS for LPS. The presenting symptoms were reported in most patients (86.5%). The latter percentage is in line with the clinical manifestations in the nationwide study in Iceland, which described only 9.5% of asymptomatic patients [14]. In our series, only 399 cases with incidental LPS were subject to RIRS. Based on the current literature, the AUA recommends conservative management as a valid option for asymptomatic lower pole stones [3]. The European Association of Urology (EAU) extends this recommendation to patients with LPS up to 15 mm [15]. Glowacki et al. evaluated the outcome of asymptomatic LPS and estimated the risk of a symptomatic stone episode or need for intervention to be approximately 10% per year with a cumulative 5-years event probability of 48.5% [16]. In a prospective study evaluating asymptomatic LPS, pain manifested during follow-up, stone size increased, or the need for intervention occurred in 11% of cases [17]. Consequently, conservative management may be a valid option for patients with LPS without any clinical manifestation, but requiring surveillance [15] and, consequently, some urologists may consider intervention in these patients.

In our analysis, 2292 (77.8%) patients resulted stone-free after the first session of lithotripsy by RIRS. This rate is similar to a multi-center randomized-controlled trial comparing SWL vs RIRS vs PCNL for single LPS of 1–2 cm [18]. In the latter, SWL showed the lowest SFR at 61.8%, while RIRS and PCNL had similar rates (82.1% and 87.3%, respectively).

Patients with intermediate (10–20 mm) sized LPS have up to a 2.25-fold higher risk of residual stones after RIRS due to the unfavorable anatomy that limits spontaneous passage, requiring a further stage for SFR frequently [19]. Several predictive factors can affect the success of surgical treatment for LPS. Indeed, variables, such as stone volume and density, infundibular length, and infundibular-pelvic angle, significantly influence the SFR. Moreover, Ito et al. showed that the renal calyxes’ size and volume affect URS success, regardless of the site of the stone [20]. In our series too, stone size was associated with higher odds of RF. Conversely, pre-stented patients showed a 75% lower chance of having RF and this was in line with a recent meta-analysis, showing that pre-stenting before RIRS was associated with higher overall SFR for < 4 and < 1 mm RF [21].

According to our analysis, TFL decreased the risk of having RF after surgery. Its flat-top pulse shape has a lower power peak and higher duration, determining a lower stone retropulsion and a higher lithotripsy efficiency through the smaller fragments’ generation. TFL has a discharged laser beam with a wavelength of 1940 nm and a fivefold water absorption than holmium laser. In addition, TFL laser fibers are more capable of resisting important ureteroscope deflections than the holmium laser, working in a pulsed or continuous mode making the laser beam more uniform and focused [22]. The greatest advantage of TFL is its versatility in range parameters (pulse energies 0.025–6 J, frequencies up to 2400 Hz, and peak power of 500 W), allowing a low and protracted peak power associated with longer pulse duration [22]. This converts in delivering more energy to the stone with lower retropulsion and thinner fragments compared to holmium laser at both fragmentation and dusting mode (fragments < 1 mm), with two-to-four times less retropulsion compared with high-power holmium laser [23]. This may indeed be an advantage for LPS where the space for lithotripsy is limited and fine dust could be better passed spontaneously.

In our study, the use of reusable scopes was significantly associated with RF. Technological development led to the improvement of the reusable scope in terms of vision due to the use of a digital camera and maneuverability. However, in vitro comparison of the single-use and reusable flexible ureteroscopes demonstrated that the formers outperformed for optical resolution, the field of view, deflection capacity, and irrigation flow with laser fiber inside the working channel [24]. This results in improved ease of use and RF reduction, as demonstrated by Yang et al., who reported higher rate of 1-month SFR in patients who underwent RIRS with disposable scopes [25]. Reusable ureteroscopes tend to perform worse over time due to working channel damage from laser burn or instrument passage and reduction of tip deflection. Therefore, based on Martin’s cost–benefit analysis, single-use scopes may be cost–beneficial in institutions with a lower volume of cases per year [26].

Among the 2946 patients of our study, 383 (12.9%) overall postoperative complications were recorded, and only 33 (1.1%) were major ones. All of them were sepsis requiring ICU admission, which is the most critical complication, and the latter is in line with the literature rate of less than 5%, as suggested by the evidence [27].

Our study has some limitations. First, its retrospective nature with bias to patient enrollment. Second, stone relocation or in situ lithotripsy is not available, although the former did not demonstrate a higher SFR compared to in situ laser lithotripsy in a recent randomized study [28]. Third, pelvicalyceal anatomy was not measured using Elbahnasy’s method [29], and we could not assess its role in SFR. Finally, the presence of RF was assessed by CT scan in only 32.5% of patients, making SFR evaluation inhomogeneous and SFR could have been more robust if postoperative CT imaging was used to verify this outcome in all patients. While CT may be the suggested evaluation standard for RF, it is difficult for all urologists to emulate this in a real-world practice due to costs, accessibility, and partly because the combination of Ultrasound and X-ray is still a common way of managing follow-up. Further, specifically for alone LPS, a CT follow-up will not increase or alter the pick-up of RF, and there is yet a dilemma of what is the ideal RF size to say that this is a clinically insignificant RF. Perhaps, this is why our multicentre cohort did not have a uniform reporting of RF. Moreover, more than half of the patients with kidney stone disease are exposed to high doses of radiation during investigation treatment and follow-up [30].

Therefore, imaging alternatives to CT could be considered to avoid unnecessary scans and the consequences of excessive radiation exposure.

## Conclusion

Despite its limitations, to the best of our knowledge, the FLEXOR study is the first large volume real-world global multi-center data that undeniably validate RIRS for LP stones as a safe and efficacious procedure and clearly shows that disposable scopes, pre-stenting, and TFL are key elements to a successful surgery. Patients with large or multiple LPS require a follow-up to determine if a second intervention is needed or, perhaps, PCNL can be considered the right choice in these patients.

## Supplementary Information

Below is the link to the electronic supplementary material.

Supplementary file1 (DOCX 13 KB)

## Data Availability

Data are available on request from the authors.
