# Supplementary material for: The efficacy of retrograde intra-renal surgery (RIRS) for lower pole stones: results from 2946 patients
Source: World J Urol. 2023 Mar 17;41(5):1407–13. doi: 10.1007/s00345-023-04363-6 (PMC10188567; doi:10.1007/s00345-023-04363-6)
Supplement: Supplementary file 1 — Supplementary file1 (DOCX 13 KB) [file 345_2023_4363_MOESM1_ESM.docx]

**Supplementary Table 1**: Postoperative parameters of patients with lower pole stones. Data are presented as frequencies (proportions).

| **Characteristics** | **Numbers** |
| --- | --- |
| Postoperative stay, *days* | 3.55 (3.38) |
| Postoperative Complications  *Fever/urinary Infections requiring antibiotics (Clavien grade 2)*  *Hematuria requiring blood transfusions (Clavien grade 2)*  *Sepsis requiring ICU admission (Clavien Grade 4)* | 169 (5.7%)  181 (6.1%)  33 (1.1%) |
| Post-operative imaging assessment by  *CT scan*  *X-ray*  *Ultrasound*  *Combination* | 956 (32.5%)  1364 (46.3%)  1493 (50.7%)  1031 (35.0%) |
| Residual fragments | 654 (22.2%) |
| Residual fragment subsequent treatment (n=654)  *SWL*  *RIRS*  *PCNL*  *ECIRS*  *Observation alone* | 132 (20.2%)  231 (35.3%)  24 (3.7%)  11 (1.6%)  256 (39.2%) |
